# Thermally Responsive Alkane Partitions and a Magnetofluidic Assay for Point-of-Sample Detection of Viruses in Wastewater

**DOI:** 10.3390/bios15050276

**Published:** 2025-04-29

**Authors:** Miso Na, David J. Boegner, Micaela L. Everitt, Ian M. White

**Affiliations:** Fischell Department of Bioengineering, University of Maryland, College Park, MD 20742, USA

**Keywords:** point-of-care, magnetofluidics, wastewater, SARS-CoV-2, COVID-19

## Abstract

Detecting, identifying, and tracking genetic material in wastewater allows public health agencies to accurately monitor the spread of infectious diseases in communities. In response to the COVID-19 pandemic, viral diagnostics for wastewater have been used to track the spread of SARS-CoV-2 and other viruses and have allowed public health officials to make more informed decisions regarding public safety. However, due to the cost and complexity of viral RNA/DNA detection platforms, analysis is limited to sophisticated laboratory facilities, which limits deployment and delays results. In contrast, a low-cost rapid point-of-sample solution for the detection of viruses in wastewater would enable worldwide deployment with immediate analytical results. We have recently reported the development of thermally responsive alkane partitions (TRAPs) for automated magnetofluidic assays, enabling sample-to-answer point-of-care detection of viruses in complex samples. Here we demonstrate the use of TRAPs in combination with hydrogel-coated magnetic particles for virus purification and assay automation to enable detection of SARS-CoV-2 from spiked wastewater samples in a low-cost cassette within a handheld instrument. Using this system, we show distinguishable detection of SARS-CoV-2 below 200 copies/mL in wastewater.

## 1. Introduction

Surveilling wastewater for genetic material from pathogens allows public health officials to track the epidemiology of infectious diseases in order to guide policy decisions. Relying on patient testing data alone can lead to an underestimate of total infections, particularly if tests are not readily available or if testing results are not being reported to public health institutions [1]. Conversely, testing wastewater samples provides a holistic representation of the spread of a pathogen through a population. Surveilling wastewater has already been used to track community spread of viral diseases such as polio [2], hepatitis [3,4], and norovirus [5] (a thorough list of pathogens that have been tracked around the globe using wastewater surveillance is presented in Kilaru et al. [6]). Most notably, in response to the COVID-19 pandemic, viral RNA detection tools for wastewater were used successfully to track SARS-CoV-2 [7] and allowed public health officials to make informed decisions.

Wastewater monitoring has been successful for tracking the spread of COVID-19 because those infected with SARS-CoV-2 shed the virus in wastewater, even in asymptomatic cases [8]. The existence of detectable virus in wastewater is attributed to SARS-CoV-2 infections in the gastrointestinal tract and eventual excretion, which can later be detected in wastewater samples for up to several days [9]. For instance, SARS-CoV-2 has been detected in wastewater of infected populations at loads of 10^2^–10^10^ copies per gram of wastewater [9,10,11]. Additionally, it has been demonstrated that wastewater samples positive for viral RNA correlate well with nasal swabs positive for viral RNA [7], making wastewater monitoring a viable alternative to individual testing to track community spread of viral diseases.

Currently, wastewater testing for viral DNA or RNA is performed with sophisticated PCR or RT-PCR tests, respectively. Wastewater samples are collected and delivered to centralized testing facilities, where they are analyzed with expensive benchtop systems. In urban areas of economically privileged regions, this model may result in a delay until results are available. More consequentially though, in rural areas and in low- and middle-income countries (LMICs), testing facilities may not even exist, implying that wastewater surveillance is not an option. Without proper data, these communities are unable to identify and track outbreaks, and thus cannot make informed public health decisions to protect the population. To deliver wastewater surveillance capabilities to these regions, it is necessary to develop a low-cost, portable, easy-to-use system that can be utilized at or near the point of sample.

One significant advancement towards point-of-care nucleic acid amplification tests has been the development of isothermal assays, which do not rely on thermal cycling for amplification as PCR does. This enables the use of simplified, lower-cost instrumentation, and in some cases, even instrument-free, electricity-free operation [12]. The primary approach to isothermal amplification has been loop-mediated isothermal amplification (LAMP), which relies on loop-forming primers to exponentially amplify target DNA, instead of cycling temperatures [13]. Just like traditional nucleic acid amplification, reverse transcriptase (RT) has been incorporated into LAMP in order to detect RNA [14,15,16]. Another notable example of isothermal amplification is recombinase polymerase amplification (RPA) [17], which relies on primer–recombinase complexes to form and exchange with the target DNA, allowing DNA polymerase to extend a primer along the target. As with LAMP, RT has been incorporated into RPA to detect RNA targets [18,19]. Another technological advancement that enhances LAMP and RPA is the use of clustered regularly interspaced short palindromic repeats (CRISPR) and CRISPR-associated proteins (e.g., Cas9, Cas12, Cas13, etc.). CRISPR-Cas schemes generally provide a fluorescence read-out based on specific recognition of the LAMP or RPA target. Numerous reports in the literature have demonstrated CRISPR-Cas schemes coupled with LAMP and RPA [20,21,22]. Generally, CRISPR-Cas is more easily coupled with RPA because the higher temperature of LAMP can inactive the components of the CRISPR-Cas readout.

Despite the advancements in nucleic acid amplification for portable detection, significant challenges remain for point-of-care and point-of-sample detection of pathogens in complex samples, including wastewater. The current preparation for wastewater samples [23] first involves filtering the sample to remove large particulates, and then lysing the viruses within the sample using a lysis buffer. To purify the RNA or DNA, solid phase adsorption onto magnetic silica microbeads is performed, typically in the presence of an alcohol to promote nucleic acid precipitation. Following this, the recovered beads must be washed to remove nucleases and proteases, and then the purified nucleic acids are eluted from the magnetic beads into an aqueous solution. A precise volume is then transferred to a PCR or RT-PCR reaction for amplification and detection. These precise steps must be performed by robotics or well-trained technicians in a sophisticated laboratory. Moreover, because viruses in wastewater are dilute, the initial sample volume needs to be on the scale of milliliters, further increasing the difficulty. New technologies are needed to mimic these steps within low-cost portable diagnostic tools.

Recently, Sharma et al. reported a simplified protocol for detection of viruses in wastewater [24]. Specifically, hydrogel-coated magnetic particles capture virus particles from pre-treated wastewater, after which the beads are removed from the wastewater, the virus is lysed via high heat, and the lysate is added to an isothermal RNA amplification reaction. While the total number of steps is reduced as compared to the conventional protocols, several precise sample manipulation steps are necessary, including spinning the sample at 5000 g, as well as sample/reagent transfer steps via micropipette.

Microfluidics is often thought of as an approach to eliminate manual steps. However, integrating multiple steps, including sample preparation and nucleic acid amplification into a single chip, requires peripheral equipment for control and expensive fabrication, and the chips are generally not compatible with real-world sample volumes. Thus, typically initial sample processing steps are performed off-chip and the amplification reaction and detection are performed on-chip. There are several examples of wastewater analysis in which lysis and nucleic acid extraction are performed off-chip and RT-LAMP or RT-RPA is performed on-chip [25,26,27,28]. While these reports have advanced the field as compared to benchtop thermal cyclers for analysis, there remains a need for a system that enables wastewater analysis in the field.

An alternative for automating nucleic acid amplification tools is magnetofluidics. In the initial reports, ferrofluids were manipulated with magnets underneath a device surface to transport droplets of reagents along the top of a surface, enabling automatic sample preparation for nucleic acid amplification [29,30,31,32,33]. Magnetic micro- or nanoparticles are used to bind nucleic acids from samples and to retain them during washes, while external magnets manipulate the beads through reagent exchanges. In the initial reports on magnetofluidic devices, the reagents were typically not enclosed and thus were susceptible to contamination, evaporation, and mechanical disturbance. More recently, some reports have demonstrated enclosed magnetofluidic assays for virus detection that utilize oil partitions between reagent regions [34,35,36,37,38]. These systems are more practical than the initial magnetofluidic approaches, although integrating oil partitions into microfluidics may be challenging at high manufacturing scale, and oil partitions may not be sufficiently robust to tolerate shipping and use in the field.

These challenges can be addressed by using phase-changing alkanes (i.e., solid alkanes that can be changed to liquid on demand) instead of oils. Recently, we reported a point-of-care sample manipulation method using pseudo-valves engineered from higher-order phase-changing alkanes to automate assays [39,40]. We have further demonstrated that these thermally responsive alkane partitions (TRAPs) possess two employable behaviors for sample-to-answer assay development [41]. They can act as (1) removeable partitions for temperature-triggered reagent addition and mixing when melted [42], and (2) stationary partitions that continually separate assay regions while allowing magnetic beads to be pulled through the melted TRAP, thus enabling simple and entirely automated magnetofluidic assays. This behavior is controllable through the geometry in which the TRAP is confined. Using TRAPs incorporated into easily-manufactured cassettes, we have reported the sample-to-answer detection of SARS-CoV-2 in blood [43] as well as in saliva samples [44].

**Figure 1:**
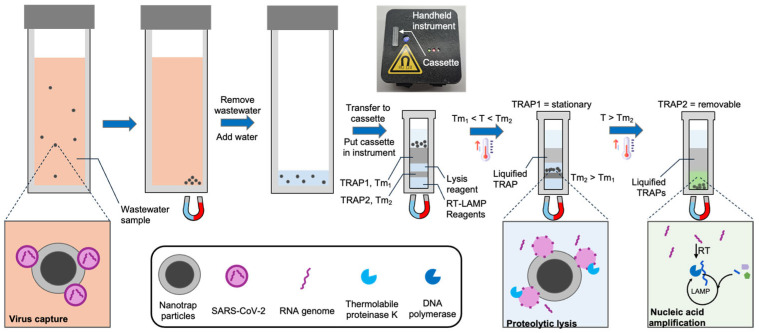
Detection of virus in wastewater using a handheld instrument, a cassette with integrated TRAPs, and hydrogel-coated magnetic particles (Nanotrap particles). The Nanotrap particles are added to 3 mL wastewater samples and briefly incubated. Following magnetic isolation, the particles are transferred to a custom cassette containing TRAPs, and the cassette is loaded into a handheld instrument. The cassette is warmed to melt the first TRAP, which liquifies but remains in place. A magnet beneath the cassette pulls the beads into a lysis reagent. After incubation, the cassette is further heated to melt the second TRAP, which breaches, causing the RT-LAMP reagents to be added to the lysate. During RT-LAMP, the instrument records the fluorescence in real time.

In this work we introduce and demonstrate a new system and workflow (Figure 1) for the detection of viruses in wastewater without the need for sophisticated instrumentation or a centralized lab. We use hydrogel-coated magnetic particles (CERES Nanotrap^®^) to non-specifically capture viruses from wastewater. These particles enable agnostic capture of a broad range of virus particles, as well as the facile purification and enrichment of captured viruses. We utilize the Nanotrap particles to capture SARS-CoV-2 from 3 mL wastewater and transfer them into a custom millimeter-scale TRAP-enabled cassette (the TRAP-enabled cassette does not require a specific volume, so an imprecise transfer pipette suffices). The cassette contains preloaded reagents for viral lysis and RNA amplification initially separated by two TRAPs. First, the cassette is heated above the melt temperature of the first TRAP, and a magnet below the cassette pulls the Nanotrap particles through a stationary TRAP into a reagent layer containing thermolabile proteinase K, which releases the RNA from the virus. The protease is sequestered from the RT-LAMP reagents using a removable TRAP. After lysis, the cassette is heated above the melt temperature of the removable TRAP, causing it to breach, which mixes the released RNA with the RT-LAMP reagents (proteinase K is heat-inactivated before the TRAP melts). The RNA is then detected using RT-LAMP. Using this approach, along with a handheld instrument, we are able to reliably detect below 200 copies/mL of wastewater, which is sufficient according to reports that SARS-CoV-2 is present at loads of 10^2^–10^10^ copies per gram of wastewater [9,10,11] for infected populations.

## 2. Materials and Methods

### 2.1. Materials

0.2 µm syringe filters were purchased from Thermo Fisher Scientific (Waltham, MA, USA). Glass vials (3.7 mL) with screw caps were purchased from VWR (Radnor, PA, USA). The alkanes used in this work include n-eicosane 99% (melt temperature ~37 °C) and n-hexacosane 99% (melt temperature ~57 °C), both sourced from Thermo Fisher Scientific. Nanotrap Microbiome A particles were acquired from CERES NANO (Manassas, VA, USA). Pierce^TM^ Streptavidin Magnetic Beads were purchased from Thermo Fisher Scientific. Antarctic Thermolabile Uracil DNA Glycosylase (UDG) was obtained from New England Biolabs (NEB, Ipswitch, MA, USA) and used throughout this work to reduce the risk of contamination during DNA amplification by removing the uracil-containing nucleic acid products. WarmStart^®^ Multi-Purpose RT-LAMP Master Mix with UDG, LAMP fluorescent dye, and thermolabile proteinase K were also purchased from NEB. The QIAamp Viral RNA Mini Kit from QIAGEN (Hilden, Germany; Germantown, MD, USA) was used for sample prep validation. Bovine serum albumin (BSA) was purchased from Sigma-Aldrich (St. Louis, MO, USA). Influenza virus A/PR/8/34 (H1N1) was supplied by ATCC (Manassas, VA, USA). Heat-inactivated SARS-CoV-2 (USA-WA1/2020) was obtained from BEI Resources (Manassas, VA, USA). *E. coli* DH5α was purchased from NEB. All DNA sequences were synthesized by Integrated DNA Technologies (IDT, Coralville, IA, USA). The sequences for the aptamer and LAMP primers are provided in the Supplementary Materials.

### 2.2. STAT Instrument and Cassette

**Figure 2:**
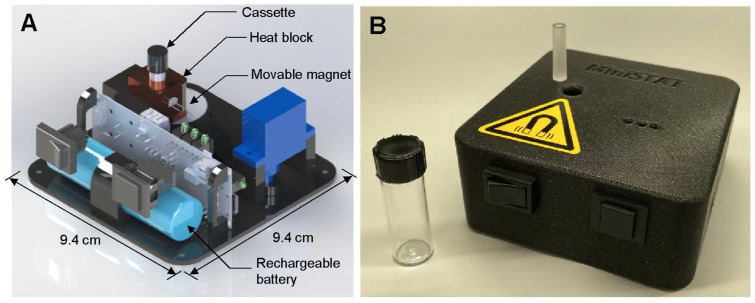
(**A**) Rendering of STAT instrument. (**B**) Photo of STAT instrument with cassette (on instrument) and glass vial.

A custom-built sample-to-answer-test (STAT) instrument was used to control the assay and measure fluorescence from the LAMP reaction. A rendering of the instrument, as well as a photo of the instrument and the assay cassette are presented in Figure 2. The STAT instrument (9.4 × 9.4 × 4 cm) includes a heater, a movable magnet, the optics for fluorescence detection, and a 2200 mAh LiPo battery capable of running multiple consecutive assays. The instrument is described in detail in our previous work [44]. A cost breakdown is provided in that work as well; at a moderate scale, the instrument could be produced for approximately USD 100. The cassettes were fabricated using computer numerical control (CNC) with an acrylic sheet (24 mm thickness; McMaster-Carr (Elmhurst, IL, USA) into 6.35 mm diameter cylinders, each with a channel 3.2 mm in diameter and 23 mm deep. The cassettes can be easily mass-produced and are expected to be comparable in cost to lateral flow assays.

### 2.3. SARS-CoV-2-Spiked Wastewater Samples

Wastewater samples were collected from wastewater pumping stations and wastewater treatment plants (WWTPs) in Maryland, USA. Pumping station samples were obtained from Arcola, Sligo Creek, Wexford, King Farm, Hoyles Mill, and Reddy Branch. Wastewater treatment plant samples were collected from Parkway, Piscataway, Western Branch, Seneca, and Damascus. Samples were collected as 24 h flow-dependent composite samples with a total volume of 500 mL. Immediately following the collection, samples were transported on ice to the laboratory at the University of Maryland for processing; samples were pooled together. Upon arrival, the samples were thoroughly mixed to ensure homogeneity. Samples were stored at 4 °C until use. SARS-CoV-2-spiked wastewater samples were prepared by filtering the sample (following virus addition) using a 1 mL syringe with a 0.2 µm pore-sized membrane. Pooled wastewater samples tested negative for SARS-CoV-2 in 100% of unspiked tests, indicating that by the time of sample use, minimal or no intact SARS-CoV-2 virus was present in the samples.

### 2.4. Validation of Viral Capture from Wastewater

To assess the efficiency of viral capture from wastewater, we compared the performance of Nanotrap hydrogel-coated magnetic particles and aptamer-functionalized magnetic beads. The aptamer-functionalized beads were prepared by incubating 25 nM of biotinylated aptamer with 100 µg of streptavidin-coated magnetic beads in 2 µL of PBS for 15 min at room temperature. Nanotrap particles were prepared simply by removing the storage buffer from 20 μL of particles before addition to samples. SARS-CoV-2-spiked wastewater samples were prepared by adding 100,000 copies of heat-inactivated SARS-CoV-2 to 1 mL of wastewater (SARS-CoV-2 stocks were quantified by the supplier and were not processed in any way before spiking them into samples). Then, 100 µL of the filtered wastewater samples were incubated with either Nanotrap particles or aptamer-functionalized beads for 30 min at room temperature. Following incubation, the beads or Nanotrap particles were pulled down with an external magnet, the supernatant was removed, and the beads/particles were resuspended in 560 µL of Qiagen AVL buffer for RNA extraction using the Qiagen RNA extraction kit. After a 10 min incubation at room temperature to lyse the virus particles, the beads were pulled down with a magnet and the supernatant was transferred to a collection tube. Next, 560 µL of 99% ethanol was added, and the RNA extraction procedure was completed according to the Qiagen instructions, with final elution in 40 µL of DI water.

To detect the extracted RNA, RT-LAMP was performed in a benchtop qPCR (MiniOpticon, Bio-Rad, Hercules, CA, USA). A reaction containing 1X WarmStart^®^ Multi-Purpose LAMP/RT-LAMP 2X Master Mix with UDG, 1X LAMP Fluorescent Dye, and LAMP primers (1.6 µM FIP/BIP, 0.2 µM F3/B3, and 0.4 µM LoopF/B) targeting the N15 gene of SARS-CoV-2 was assembled. DI water was added to bring the volume to 20 µL per reaction. The mixture was vortexed and loaded into PCR tubes. Then, 5 µL of extracted RNA was added to each reaction tube. The thermocycler was set to 65 °C while continuously monitoring fluorescence to track amplification in real time.

### 2.5. Validation of Proteolytic Lysis Following Viral Capture

In our developed system, proteolytic lysis was performed using thermolabile proteinase K in the assay cassette. To validate the effectiveness of proteolytic lysis compared to the Qiagen RNA extraction kit, we conducted the following experiments. SARS-CoV-2-spiked wastewater samples were prepared and the virus was captured with Nanotrap hydrogel-coated magnetic particles or aptamer-functionalized magnetic beads in 100 µL of the sample as described in Section 2.4. Following the virus capture, the beads were pulled down, the supernatant was removed, and the pellet was resuspended in 40 µL of DI water. An amount of 1 µL (0.12 units) of thermolabile proteinase K was added to the resuspended sample. The sample was then incubated in a thermocycler at 37 °C for 10 min, followed by heat inactivation at 55 °C for 15 min. The lysed sample was subsequently analyzed with RT-LAMP as described in Section 2.4.

### 2.6. Detection of SARS-CoV-2 in TRAP-Enabled Cassettes Using Hydrogel-Coated Magnetic Particles

The assay cassettes were preloaded with two alkane TRAP layers, a lysis solution, and a LAMP reaction solution. The LAMP reaction was prepared by combining 12.5 µL WarmStart^®^ Multi-Purpose RT-LAMP 2X Master Mix with UDG (NEB), 0.5 µL 50X LAMP Fluorescent Dye, and 2 µL DI water. The lysis solution was prepared by combining 1 µL thermolabile proteinase K, 2.5 µL LAMP primer mix (1.6 µM FIP/BIP, 0.2 µM F3/B3, and 0.4 µM LoopF/B) targeting the N15 gene, 1 µL Antarctic Thermolabile UDG, and 6 µL DI water. First, 15 µL of LAMP reaction solution was added to the bottom of the cassette, followed by 10 µL of hexacosane. After the hexacosane solidified, 10 µL of the lysis solution was added, followed by 35 µL of eicosane. Finally, magnetic particles (after incubation with the sample) were loaded into the top layer of the cassette (Figure 1). The cassette was then placed in the STAT instrument for temperature control and for fluorescence-based measurements.

In the STAT instrument, the cassette was heated to 39 °C for 6 min to melt the first TRAP (eicosane). Once it melted, the magnetic beads were automatically pulled through the eicosane layer into the lysis layer by a magnet positioned beneath the cassette throughout the assay. The STAT instrument then decreased the temperature to 35 °C for 6 min for proteolytic lysis. Then, the temperature was increased to 49 °C for 5 min to inactivate the thermolabile proteinase K. Finally, the temperature was increased to 60 °C, melting the second TRAP layer (hexacosane) and allowing the LAMP reagents to mix with the lysate, initiating the amplification reaction. The LAMP reaction proceeded at 60 °C, and fluorescence was continuously recorded by the STAT instrument. Temperature control is fully automated by the STAT instrument.

To assess assay tolerance to interfering elements, we conducted target virus capture, purification, and amplification experiments in the presence of potential interfering substances: BSA, *E. coli*, and H1N1 influenza virus. First, 50 µL of Nanotrap particles were prepared by removing the storage solution. For virus capture, the beads were combined separately with the following mixtures: 100 µL PBS containing 1 mg/mL BSA and 5000 copies of SARS-CoV-2, 100 µL PBS containing 10^6^ CFU of *E. coli* and 5000 copies of SARS-CoV-2, and 100 µL PBS containing approximately 60,000 copies of H1N1 influenza virus and 5000 copies of SARS-CoV-2. Each mixture was combined with the prepared beads and incubated for 30 min at room temperature. Negative controls were prepared identically but without the SARS-CoV-2 virus.

### 2.7. Detection of SARS-CoV-2 in Wastewater Samples with the STAT Instrument

To evaluate the detection of SARS-CoV-2 in wastewater samples using the STAT instrument, we tested 3 mL of SARS-CoV-2-spiked wastewater samples. Virus capture was achieved by incubating 100 µL of Nanotrap particles (after removal of the beads’ storage buffer and resuspension in 50 µL of DI water) with the 3 mL sample for 30 min at room temperature. Mixing was performed by loading the sample and beads into a glass vial (Figure 2B), closing the lid securely, and shaking vigorously by hand for 10 s. After 15 min, the vial was shaken again using the same method. Following the incubation, the glass vial was placed on a magnet, and the wastewater was removed. The remaining beads were resuspended in about 200 µL of deionized (DI) water and divided into two cassettes, which were analyzed in parallel. Then, the resuspended magnetic particles were transferred into the cassette preloaded with lysis solutions and LAMP, and the assay was performed using the STAT instrument as detailed in Section 2.6.

## 3. Results and Discussion

### 3.1. Hydrogel-Coated Magnetic Particles Effectively Capture Viruses from Wastewater

**Figure 3:**
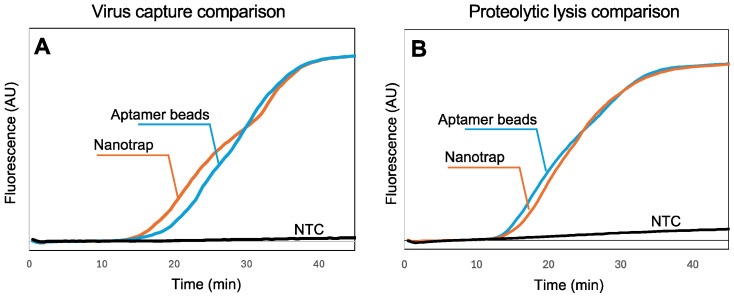
(**A**) Real-time fluorescence measurements showing RT-LAMP amplification of SARS-CoV-2 RNA following viral capture in 100 μL wastewater and standard sample processing (10,000 viral copies). Normalized amplification curves are the average of N = 3. (**B**) Real-time fluorescence measurements showing RT-LAMP amplification of SARS-CoV-2 RNA following lysis using thermolabile proteinase K (10,000 virus copies). All amplification curves are provided in the Supplementary Materials. Normalized amplification curves are the average of N = 4. The normalization procedure for all curves is provided in the Supplementary Materials.

In our previous work with SARS-CoV-2 [43,44], we utilized aptamer-functionalized magnetic beads to capture the virus in biological samples. However, aptamers are vulnerable to nucleases in sample matrices, they require special storage conditions, and they are specific to one virus. In contrast, the chemistry of the Nanotrap hydrogel-coated magnetic particles is not affected by nucleases, they can be stored at room temperature, and they are not specific to a single virus, suggesting the possibility of multiplexing. To assess the capability of the Nanotrap particles to capture SARS-CoV-2 virus in wastewater samples, we added Nanotrap particles or aptamer-functionalized microbeads to spiked wastewater samples with SARS-CoV-2, then recovered the beads and used a standard RNA extraction kit followed by RT-LAMP in a benchtop qPCR to quantify the amount of virus captured. Figure 3A shows the real-time amplification curves recorded by the thermal cycler. Amplification is evident within about 15 min, demonstrating that the Nanotrap particles can capture SARS-CoV-2 from wastewater and release the RNA following lysis.

### 3.2. Hydrogel-Coated Magnetic Particles Are Compatible with Proteolytic Lysis

While Figure 3A demonstrates that the Nanotrap magnetic particles are compatible with the standard RNA extraction kit, it is difficult to implement these steps into a sample-to-answer workflow because of the need for multiple rinses and spins. In our previous work [43,44], we demonstrated that thermolabile proteinase K followed directly by RT-LAMP (without reagent exchange or washing) can be used to implement a sample-to-answer workflow for the detection of SARS-CoV-2. However, because the Nanotrap particles capture viruses within a nanoporous hydrogel network, accessibility of the virus to proteinase K and release of the RNA needed to be investigated. We added Nanotrap particles or aptamer-functionalized magnetic beads to spiked wastewater samples with SARS-CoV-2, then recovered the beads/particles and added proteinase K. After incubation and heat inactivation, we transferred a portion of the lysed sample to an RT-LAMP reaction and amplified it using a benchtop qPCR (Figure 3B) to validate the RNA release. The amplification reactions with the two particle chemistries show very similar times-to-positive, indicating that the Nanotrap particles are compatible with a proteolytic-based virus lysis. These results suggest that lysis with thermolabile proteinase K can be effectively integrated into our simplified workflow with the hydrogel-coated magnetic particles without compromising detection performance.

### 3.3. Hydrogel-Coated Magnetic Particles Are Compatible with TRAPs

**Figure 4:**
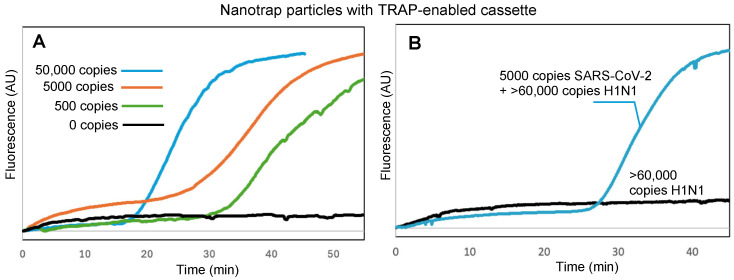
(**A**) Real-time fluorescence measurements showing RT-LAMP amplification of SARS-CoV-2 RNA following viral capture by hydrogel-coated magnetic particles, transition of the particles through a stationary TRAP, proteolytic lysis, and RT-LAMP, all within the TRAP-enabled cassette. (**B**) Specific detection of SARS-CoV-2 with samples spiked with >60,000 copies of H1N1 virus. Normalized amplification curves are the average of N = 3. The normalization procedure for all curves is provided in the Supplementary Materials.

The key to our sample-to-answer workflow is thermally responsive alkane partitions (TRAPs). TRAPs enable the automated purification of captured virus from complex samples by enabling magnetic beads to be pulled through the liquified partitions while the aqueous assay reagents remain partitioned from the aqueous sample matrix. To investigate whether the hydrogel-coated magnetic particles could be pulled through a TRAP without compromising virus capture and retention, we spiked 100 μL of DI water with virus and added it to the top of a TRAP-enabled cassette (Figure 1) and placed the cassette into the handheld instrument (Figure 2) to run the assay. Figure 4A shows the real-time amplification curves recorded by the instrument (average of N = 3) for 50,000 copies, 5000 copies, 500 copies, and 0 copies of virus; time t = 0 corresponds to the time that the hexacosane layer in the TRAP cassette melted, adding the released RNA to the RT-LAMP reagents (real-time fluorescence measurements for the entire duration of the assay, along with real-time temperature measurements of the cassette, are provided in the Supplementary Materials, Figure S3).

We further confirmed that Nanotrap particles and TRAP-enabled cassettes maintain their performance in samples with competing background components. First, we spiked SARS-CoV-2 into a sample of 1 mg/mL BSA, which could compete for the surface area on the hydrogel-coated magnetic particles. Despite the high background protein concentration, amplification and detection was successful (Figure S4, Supplementary Materials). Similarly, amplification and detection was successful when virus was spiked into a sample containing 10^6^ CFU of *E. coli* (Figure S5, Supplementary Materials). To demonstrate specific detection of SARS-CoV-2, we spiked samples with H1N1 or both SARS-CoV-2 and H1N1. Figure 4B shows that amplification did not occur with H1N1 only, but did occur when SARS-CoV-2 was present. Times to positive for all three challenges were similar to those in Figure 4A for the same number of copies of SARS-CoV-2, indicating that minimal or no inhibition occurred.

### 3.4. Hydrogel-Coated Magnetic Particles Combined with TRAPs Enable Sensitive and Specific Detection of SARS-CoV-2 in Wastewater

To assess the performance of our system for the detection of viruses in wastewater, we spiked varying concentrations of SARS-CoV-2 in wastewater samples and utilized the TRAP-enabled cassette and the handheld STAT instrument with the workflow presented in Figure 1. Because viruses are dilute in wastewater, a large sample volume must be utilized. Meanwhile, a larger sample volume implies the need for a larger amount of capturing particles. We found that we could successfully detect dilute concentrations of virus in 3 mL of wastewater using 100 μL of Nanotrap particles with only a 30 min incubation. Because the TRAP-enabled cassettes are reliable with up to 50 μL of Nanotrap particles, the recovered beads were split into two separate cassettes and the detection assay was run in parallel in two low-cost handheld instruments. If either cassette showed amplification within 90 min, the assay was considered positive. If both cassettes showed no amplification within 90 min, the assay was considered negative.

The results for 5000, 500, 50, and 0 viral copies are summarized in Table 1. Perfect detection is evident for 5000 and 500 copies. For 50 copies spiked into the 3 mL sample, two of the three trials resulted in false negatives, demonstrating that this was below our limit of detection. For zero copies, all trials resulted in true negatives, indicating that the detection in our assay is specific.

## 4. Conclusions

In this study, we demonstrated an effective point-of-sample detection methodology for SARS-CoV-2 directly from wastewater using hydrogel-coated magnetic particles combined with TRAPs integrated into a low-cost and easily manufactured cassette. This method delivers results within approximately 2 h, including the 30 min virus capturing incubation period. Our results showed that hydrogel-coated magnetic particles effectively captured and concentrated SARS-CoV-2 from 3 mL of complex wastewater samples, retained the captured virus during TRAP-mediated purification, and were fully compatible with proteolytic lysis using thermolabile proteinase K. The assay incorporates virus purification, proteolytic lysis, and subsequent nucleic acid amplification in a portable, handheld instrument, achieving reliable detection at concentrations below 200 copies/mL. Validation tests confirmed the robust performance of our method, effectively distinguishing positive samples from negative samples and supporting its applicability for field applications. This low-cost, portable system is especially promising for real-time public health monitoring in resource-limited settings.

The next stage of research on phase-changing alkane partitions can focus on real-world deployment. Most notably, the work presented here utilized reagents stored in an aqueous state. However, for true field deployment, the reagents should be lyophilized in the cassette. While we previously showed that alkane partitions are compatible with lyophilization [39], assay optimization is still needed. In addition, optimal selection of alkane materials is needed. Here we used eicosane for one TRAP, but this partition might not be stable in warmer environments. Other alkanes, such as docosane, which has a melt temperature above 40 °C, can be investigated as alternatives. In other future work, we will further simplify the workflow by integrating the magnetic recovery of the virus particles and the remaining steps of the assay into a single cassette for complete wastewater-to-answer operation.

Finally, future work will aim to improve the limit of detection. In the work presented here, we were able to detect 500 total viral copies in 3 mL of sample. However, we were previously able to show that we could detect five copies of RNA in a cassette in the STAT [44]. Sources of loss include inefficiencies in viral capture, transport through the TRAP, lysis, and release of RNA from the Nanotrap particles. Increasing the sample volume, the quantity of Nanotrap particles, and the incubation time can increase the number of captured virus particles available for detection. Reagents and lysis time/temperature can be further optimized as well, though we note that surfactant concentration must be minimized (<0.1%) to maintain the alkane TRAP stability.

While the Nanotrap particles provide several advantages for virus capture and purification, we note one disadvantage as compared to immuno- or aptamer-coated magnetic particles. Wastewater can contain significant amounts of debris. Typical sample preparation workflows include a filtration step to remove large debris that could clog components during DNA/RNA recovery and to remove excessively high amounts of flora (e.g., fungi, bacteria) that could release large amounts of DNA that could interfere with viral genome recovery and amplification. However, for aptamer-coated magnetic particles and TRAPs, we do not need to perform the filtration step, as the particles will only bind to the targeted virus, and the TRAP will prevent debris/microorganisms from passing through to the DNA/RNA recovery stage. This workflow does not work as well for the Nanotrap particles because they can bind non-specifically to other components within the wastewater sample, potentially forming large aggregates that cannot pass through the TRAPs. Thus, filtration was required in this work.

One additional advantage of the Nanotrap particles not explored in this work is the opportunity for multiplexing for broad surveillance. This work focused on the detection of SARS-CoV-2 for the surveillance of COVID-19, but there is a need to track numerous dangerous viruses to monitor for outbreaks. An ideal solution would be capable of detecting multiple viruses of interest. We note that the Nanotrap particles are non-specific and thus are capable of capturing a broad range of viruses. The assay, including amplification and read-out, can be multiplexed by utilizing multiple primer sequences and by utilizing an alternative read-out strategy. While multi-color fluorescence can be used [45], this increases the cost of the equipment. Recently, lateral flow assays have been utilized to provide a multiplexed read-out from LAMP [46,47]. CRISPR-Cas12 can be used to simplify this [48,49]. These options should be investigated for further enhancements to the TRAP-based assays and STAT instrument.

## Figures and Tables

**Table 1:** Summary of results for testing the detection of virus in 3 mL wastewater samples using TRAP-enabled cassettes and the handheld STAT instrument. **+**: time to positive < 90 min; **-**: not positive within 90 min; **N**: defective cassette; **TP**: true positive; **TN**: true negative; **FN**: false negative.

Wastewater Sample	Cassette #1	Cassette #2	Result
5000 copies /3 mL	**+**	**+**	**TP**
	**+**	**+**	**TP**
	**+**	**-**	**TP**
500 copies /3 mL	**+**	**-**	**TP**
	**+**	**-**	**TP**
	**+**	**+**	**TP**
50 copies /3 mL	**+**	**N**	**TP**
	**-**	**-**	**FN**
	**-**	**-**	**FN**
0 copies /3 mL	**-**	**-**	**TN**
	**-**	**-**	**TN**
	**-**	**-**	**TN**

## Data Availability

The data presented in this study are available on request from the corresponding author.

## Supplementary Materials

The following supporting information can be downloaded at: https://www.mdpi.com/article/10.3390/bios15050276/s1, DNA sequences utilized in the experiments; Figure S1: complete data set for Figure 3A; Figure S2: complete data set for Figure 3B; Figure S3: fluorescence and temperature values recorded by the STAT for a complete experiment in Figure 4A; Figure S4: amplification curves for SARS-CoV-2 spiked into 1 mg/mL BSA; Figure S5: amplification curves for SARS-CoV-2 spiked along with 10^6^ CFU *E. coli*; Figure S6: complete data set of fluorescence values recorded by the STAT for Figure 4B; description of normalization procedures for amplification curves in Figure 3 and Figure 4. References [50,51] are cited in the Supporting Information.
