# Supplementary material for: Thermally Responsive Alkane Partitions and a Magnetofluidic Assay for Point-of-Sample Detection of Viruses in Wastewater
Source: Biosensors (Basel). 2025 Apr 29;15(5):276. doi: 10.3390/bios15050276 (PMC12109948; doi:10.3390/bios15050276)
Supplement: Supplementary file 1 [file biosensors-15-00276-s001.zip › biosensors-3572201-supplementary.pdf]

Thermally responsive alkane partitions and a magnetofluidic assay for point-of-sample detection of viruses in wastewater

Electronic Supporting Information

Miso Na[1], David J. Boegner[1], Micaela L. Everitt[1], Ian M. White[1]*

[1]Fischell Department of Bioengineering, University of Maryland, College Park, MD 20742, USA
*** Corresponding author**
ianwhite@umd.edu

DNA sequences

Virus particles were captured onto streptavidin-functionalized paramagnetic beads using biotinylated SARS-CoV-2 RBD aptamer [1]:

5'–ATCCAGAGTGACGCAGCATTTCATCGGGTCCAAAAGGGGCTGCTCGGG ATTGCGGATATGGACACGTTTTTTTT /3Bio/ – 3'.

The SARS-CoV-2 RNA genome was amplified with LAMP using the following primers for the N15 gene [2]:

FIP 5' – TGCTCCCTTCTGCGTAGAAGCCAATGCTGCAATCGTGCTAC – 3'

BIP 5' – GGCGGCAGTCAAGCCTCTTCCCTACTGCTGCCTGGAGTT – 3'

F3 5' – AGATCACATTGGCACCCG – 3'

B3 5' – CCATTGCCAGCCATTCTAGC – 3'

LF 5' – GCAATGTTGTTCCTTGAGGAAGTT – 3'

LB 5' – GTTCCTCATCACGTAGTCGCAACA – 3'

Hydrogel-coated magnetic particles effectively capture viruses from wastewater: data analysis

For the experiments presented in Figure 3A, fluorescent values were recorded in real time by the MiniOpticon qPCR system. For curves that amplified, each curve was normalized by (i) subtracting the fluorescence value at 7.5 minutes to achieve a 0 baseline and (ii) dividing all values by the fluorescence value at 80 minutes (after background subtraction). For curves that did not amplify, each curve was normalized by (i) subtracting the fluorescence value at 7.5 minutes to achieve a 0 baseline and (ii) dividing all values by the average fluorescence value at 45 minutes for all curves that amplified (after background subtraction). All curves are shown in Figure S1.

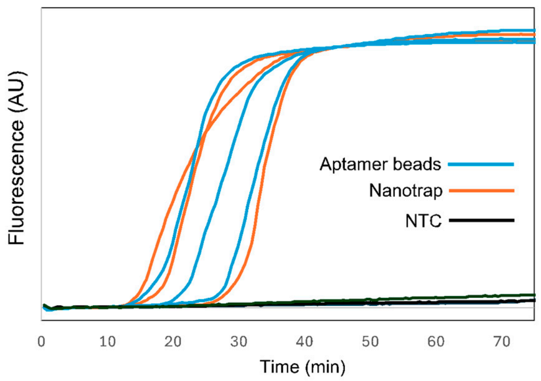

Figure S1. Real-time fluorescence measurements showing RT-LAMP amplification of SARS-CoV-2 RNA following viral capture in 100 µL wastewater and standard sample processing with the Qiagen kit.

Hydrogel-coated magnetic particles are compatible with proteolytic lysis: data analysis

For the experiments presented in Figure 3B, fluorescence values were recorded in real time by the Mini Optical qPCR system. For curves that amplified, each curve was normalized by (i) subtracting the fluorescence value at 7.5 minutes to achieve a 0 baseline and (ii) dividing all values by the fluorescence value at 80 minutes (after background subtraction). For curves that did not amplify, each curve was normalized by (i) subtracting the fluorescence value at 7.5 minutes to achieve a 0 baseline and (ii) dividing all values by the average fluorescence value at 80 minutes for all curves that amplified (after background subtraction). All normalized curves are shown in Figure S2.

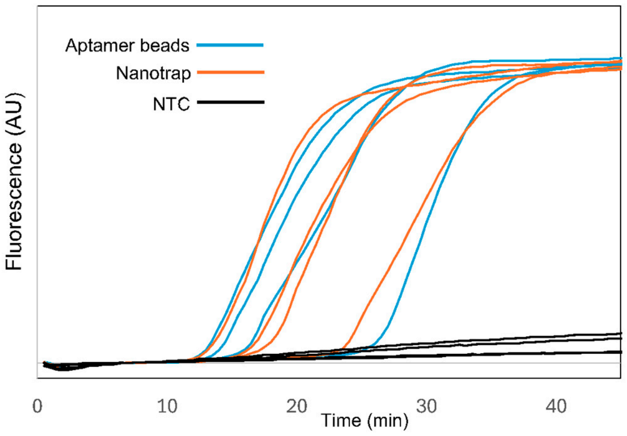

Figure S2. Real-time fluorescence measurements showing RT-LAMP amplification of SARS-CoV-2 RNA following lysis using thermolabile proteinase K.

Hydrogel-coated magnetic particles along with TRAPs enable sensitive and specific detection of SARS-CoV-2: fully automated temperature actuation

The handheld STAT instrument actuates the TRAPs using temperature control, enabling complete automation of the assay. Figure S3 shows the fluorescence intensity (raw data) and the measured cassette temperature versus time throughout the entire assay for one of the curves recorded in the TRAP-enabled cassette. The graph is labeled to indicate what step is occurring at each temperature. Notably, it is clear from the fluorescence measurements when the hexacosane melts, enabling time t = 0 of the RT-LAMP to be established.

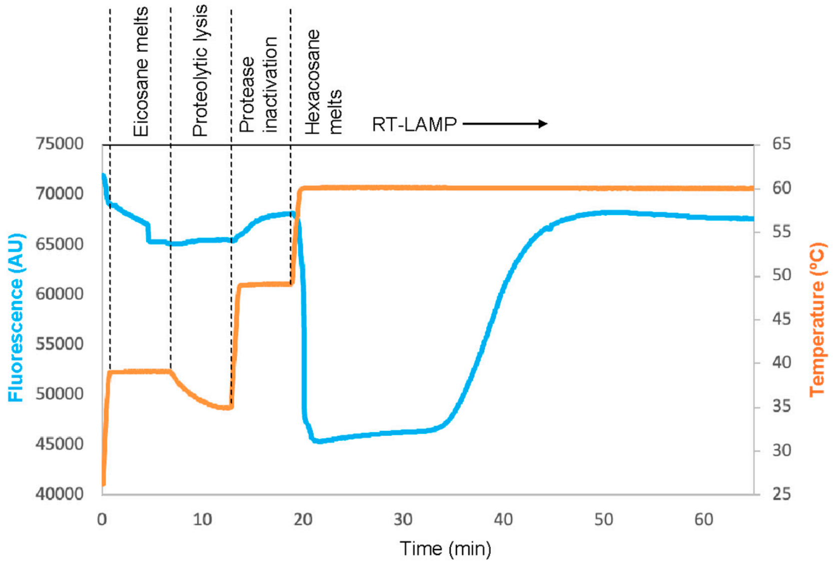

Figure S3. Real-time fluorescence measurements and cassette temperature measurements recorded by the handheld STAT instrument for an assay with a sample spiked with SARS-CoV-2.

Hydrogel-coated magnetic particles along with TRAPs enable sensitive and specific detection of SARS-CoV-2 in samples with competing elements

We demonstrated capture, purification, and amplification of SARS-CoV-2 in samples containing 1 mg/mL BSA (Figure S4) and 10^6 CFU of *E. coli* (Figure S5). We also demonstrated specific detection of SARS-CoV-2 in samples spiked with H1N1 (Figure S6). Fluorescent values were recorded in real time by three individual handheld STAT instruments. For all curves, time t = 0 was set to the time that the hexacosane layer melted. For curves that amplified, each curve was normalized by (i) subtracting the fluorescence value at time t = 0 to achieve a 0 baseline and (ii) dividing all values by the fluorescence value at reaction saturation (after background subtraction). For curves that did not amplify, each curve was normalized by (i) subtracting the fluorescence value at 0 minutes to achieve a 0 baseline and (ii) dividing all values by the fluorescence value prior to hexacosane melting.

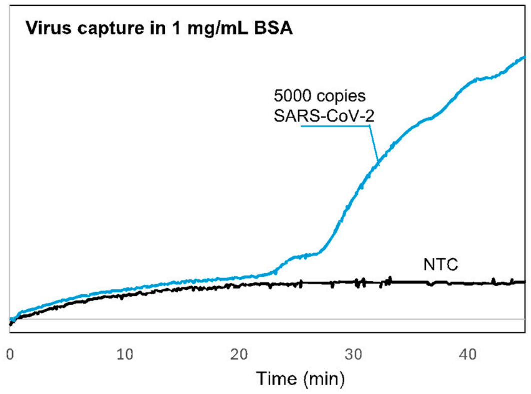

Figure S4. Real-time fluorescence measurements showing RT-LAMP amplification of SARS-CoV-2 RNA following capture of the virus by Nanotrap particles in the presence of 1 mg/mL BSA, transport through a stationary TRAP, lysis with proteinase K, and RT-LAMP, all within a TRAP-enabled cassette. Curves are the average of N=3, normalized as explained above.

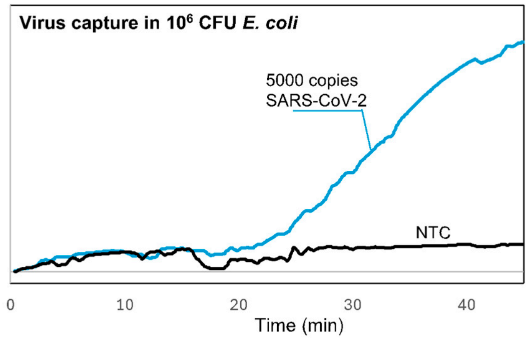

Figure S5. Real-time fluorescence measurements showing RT-LAMP amplification of SARS-CoV-2 RNA following capture of the virus by Nanotrap particles in the presence of 10^6 CFU of *E. coli*, transport through a stationary TRAP, lysis with proteinase K, and RT-LAMP, all within a TRAP-enabled cassette. Curves are the average of N=3, normalized as explained above.

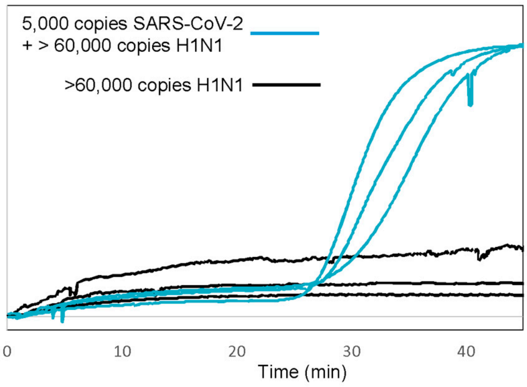

Figure S6. Real-time fluorescence measurements showing RT-LAMP amplification of SARS-CoV-2 RNA following capture of the virus by Nanotrap particles in the presence of >60,000 copies of H1N1 Influenza virus, transport through a stationary TRAP, lysis with proteinase K, and RT-LAMP, all within a TRAP-enabled cassette. Curves are normalized as explained above.

References

1. Y. Song, J. Song, X. Wei, M. Huang, M. Sun, L. Zhu, B. Lin, H. Shen, Z. Zhu, C. Yang, Discovery of Aptamers Targeting the Receptor-Binding Domain of the SARS-CoV-2 Spike Glycoprotein, *Anal. Chem*. **2020**, 92, 9895–9900.
2. W.E. Huang, B. Lim, C. Hsu, D. Xiong, W. Wu, Y. Yu, H. Jia, Y. Wang, Y. Zeng, M. Ji, H. Chang, X. Zhang, H. Wang, Z. Cui, RT-LAMP for rapid diagnosis of coronavirus SARS-CoV-2, *Microb. Biotechnol.* **2020**, 13, 950–961.
